# Dealing with perceptions related to thrombosis and COVID-19 vaccines

**DOI:** 10.26633/RPSP.2021.45

**Published:** 2021-07-08

**Authors:** Céleo Ramírez, Edwin F. Herrera Paz, Sandra Gómez Ventura, Gaspar Rodríguez, Nery Linarez, Reyna M. Durón

**Affiliations:** Hospital del Valle San Pedro Sula Honduras Hospital del Valle, San Pedro Sula, Honduras; Universidad Católica de Honduras San Pedro Sula Honduras Universidad Católica de Honduras, San Pedro Sula, Honduras; Consorcio de Investigadores COVID Honduras Honduras Consorcio de Investigadores COVID Honduras, Honduras; Hospital General del Sur Choluteca Honduras Hospital General del Sur, Choluteca, Honduras; Observatorio de COVID-19, Universidad Tecnológica Centroamericana, UNITEC Tegucigalpa Honduras Observatorio de COVID-19, Universidad Tecnológica Centroamericana, UNITEC Tegucigalpa, Honduras

To the Editor:

To date, there have been around 178 million confirmed SARS CoV-2 infections, and more than 3 million deaths worldwide. The global war against COVID-19 has been fought mainly in three battlegrounds: the hospitals, the communities, and in the minds of people. As populations around the globe still struggle to establish or keep the public health strategies needed, they also face an *infodemic*, especially regarding vaccines.

All vaccines can have rare adverse events, but during the pandemic, the report of isolated cases of blood clots associated to two of the anti-COVID vaccines that use adenovirus as a viral vector (AZD1222 and Johnson & Johnson) have raised concerns in the population, as well as emergency reviews, safety signals, and vaccination pauses (1-4).

According to several reports, thrombosis associated to the AZD1222 vaccine occurred mainly in women under 55 years of age (1,3). Some governments of high-income countries that have more than one vaccine type available, like the United Kingdom and Canada, have instructed to administer AZD1222 according to specific age groups of apparent less risk (3,4). Others, like Denmark, stopped the use of that vaccine indefinitely (5).

Thrombosis after the two aforementioned COVID vaccines seems to be more common in women but almost non-existent in older adults, who could be the best candidates to receive them (1). This context shows that no vaccine can be discarded and illustrates that decisions regarding the use of available vaccines will improve as research and pharmacovigilance continues. Individual perceptions can influence the collective decision-making. Understanding that can help to reduce vaccine hesitancy, especially the one related to frequent alerts on adverse events.

The variety of options available for some high-income countries allows them to choose some vaccines over others, and they carry out such measures without jeopardizing their mass vaccination programs. On the other side, low and middle-income countries still lack access to vaccines. At the same time, the latter are generally weak in enforcing the most basic measures to contain the virus, their hospitals collapse rapidly, and they face challenges to even provide oxygen supply for patients in public settings.

From the global health perspective, fast and massive vaccination is a priority for all countries in order to get the herd immunity that can help stop the pandemic and its consequences. About one fifth of those who suffer from COVID-19 are at risk of a severe disease, and that is enough to bring any health system in the world to its knees. One key message for the public is that the benefit of vaccination far outweighs the risks, and that efforts are being made to reduce and treat this risk. According to several studies, the frequency of any type of thrombosis in COVID-19 can be up to 17.6%(6), while initial data from the European Medicines Agency estimated a frequency of approximately 1 per 1,000,000 people vaccinated with the AZD1222 (1).

Regulatory offices and governments must keep an adequate and objective communication with citizens in order to reduce the reluctance to the application of vaccines. Developed countries have more capacity to develop research and adequate pharmacovigilance systems for the early detection and treatment of adverse events. However, all countries should get that capacity, even if international support is necessary. Therefore, the alternative is to develop adequate pharmacovigilance systems, credibility and leadership from governments that work together with the population, a strategic education plan aided by scientists, participation of the media and political leaders, and to prepare healthcare systems to assist citizens in the immediate post vaccine period.

Vaccination together with public health measures will reflect on hospital occupation and economy recovery. Reaching most of the population with any available vaccine is also a time trial against the emergence of new variants of the virus that circumvent the immunity generated by vaccines.

## Disclaimer.

Authors hold sole responsibility for the views expressed in the manuscript, which may not necessarily reflect the opinion or policy of the RPSP/PAJPH or the Pan American Health Organization (PAHO).
